# Supplementary material for: ED Formula, a Complex of Ecklonia cava and Chrysanthemum indicum, Ameliorates Airway Inflammation in Lipopolysaccharide-Stimulated RAW Macrophages and Ovalbumin-Induced Asthma Mouse Model
Source: Pharmaceuticals (Basel). 2023 Aug 21;16(8):1185. doi: 10.3390/ph16081185 (PMC10458152; doi:10.3390/ph16081185)

Original Images for Blots/Gels

Supplementary Figure S1. Original photographs for the blots of each protein marker of Figure 2(C-E).

<u>Figure 2</u>

(C)

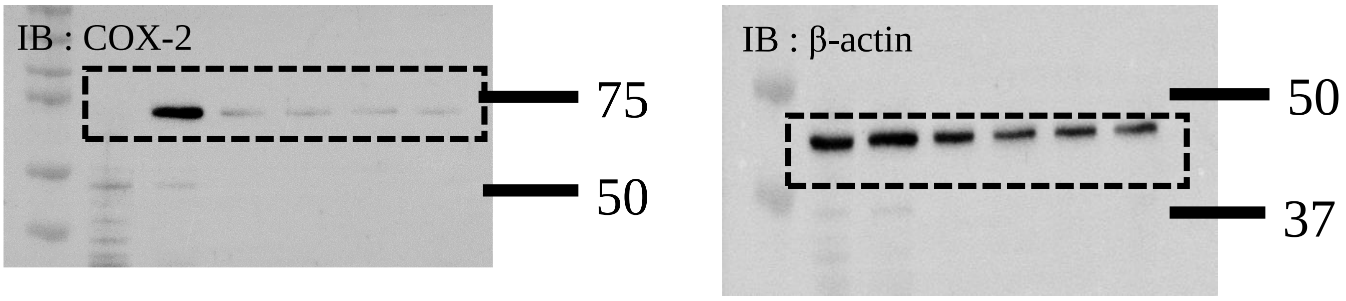

(D)

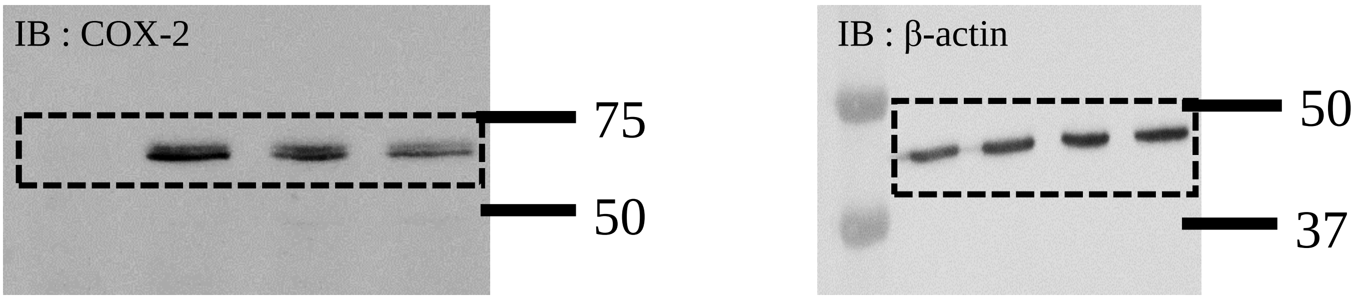

(E)

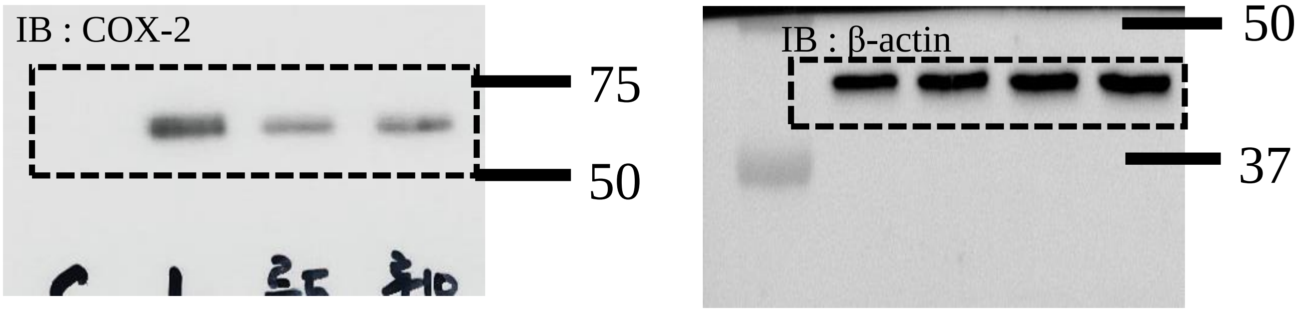

Original Images for Blots/Gels

Supplementary Figure S2. Original photographs for the blots of each protein marker of Figure 3(A-E).

Figure 3

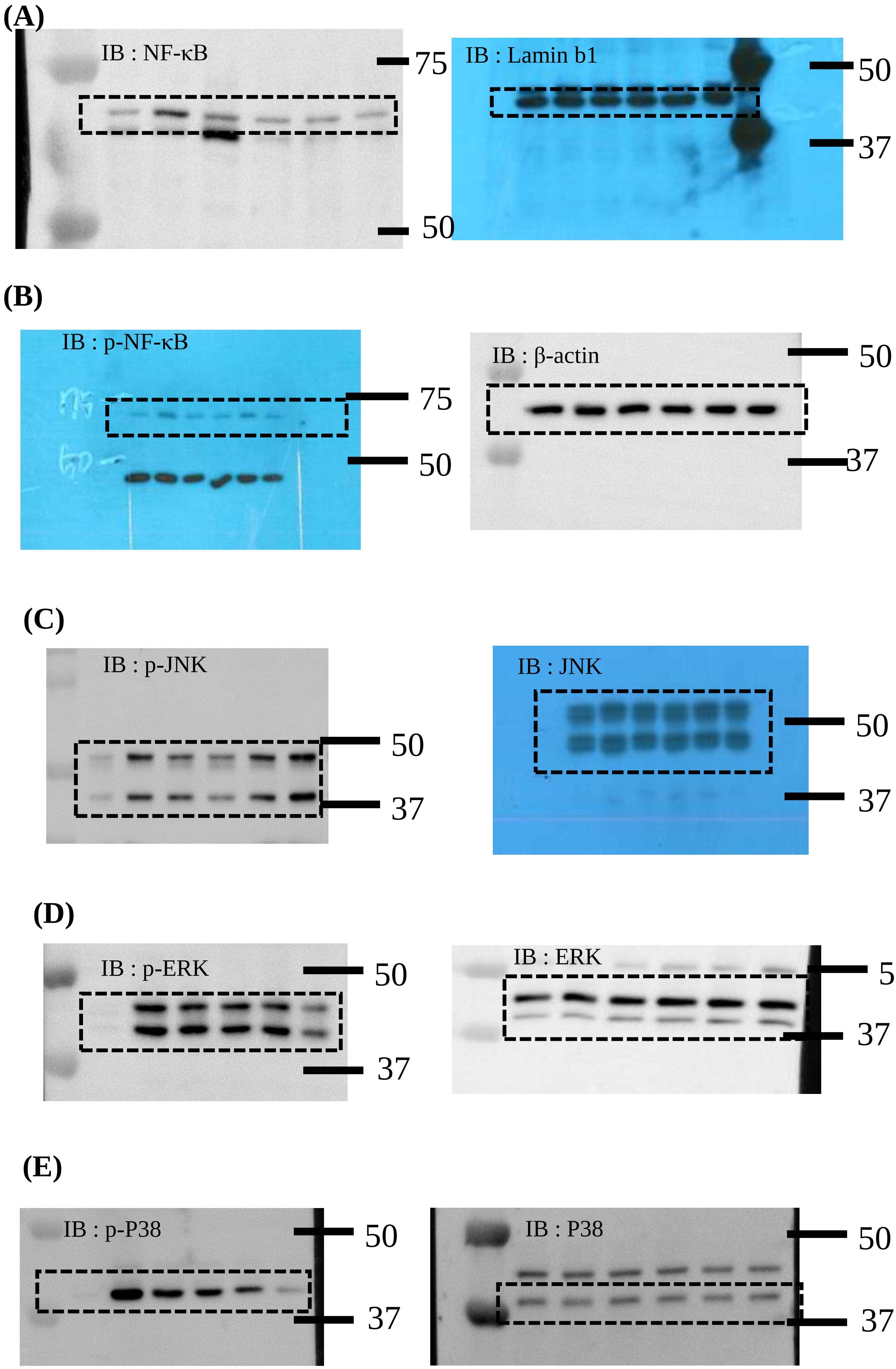

Supplement: Supplementary file 1 [file pharmaceuticals-16-01185-s001.zip › pharmaceuticals-2532236-supplementary.pdf]
